# Account of Diseases in an Eastern District of London, from October 20, to November 20, 1804

**Published:** 1804-12-01

**Authors:** 


### *Account of Diseases in an Eastern District of London, from October 20, to November 20, 1804.*

| | |
|---|---|
| ACUTE DISEASES. | |
| Typhus | 4 |
| Ephemera | 5 |
| Dysenteria | 6 |
| Variola | 2 |
| Rheumatismus Acutus | 3 |
| CHRONIC DISEASES. | |
| Catarrhus | 3 |
| Tussis | 17 |
| Dyspnœa | 15 |
| Tussis cum Dyspnœa | 20 |
| Hæmoptoe | 2 |
| Phthisis Pulmonalis | 4 |
| Hydrops Pectoris | 3 |
| Menorrhagia | 2 |
| Dysmenorrhœa | 2 |
| Amenorrhœa | 5 |
| Chlorosis | 2 |
| Diarrhœa | 20 |
| Vermes | 2 |
| Rheumatismus Chronicus | 20 |
| PUERPERAL DISEASES. | |
| Ephemera | 7 |
| Menorrhagia Lochialis | 4 |
| Mastodynia | 3 |
| Hæmorrhois | 2 |
| INFANTILE DISEASES. | |
| Aphthæ | 7 |
| Diarrhœa | 14 |
| Ophthalmia | 3 |
| Ophthalmia Purulenta | 2 |
| Herpes | 5 |

Typhus, which for a considerable time appeared very seldom, has lately resumed its place in our list of diseases. It has occurred more frequently within the last three or four months than it has for a long period; nor has it always appeared in its mildest form: In most instances the symptoms have been formidable, and in some cases the disease has proved fatal.

In addition to the usual symptoms, affections of the head have prevailed to an uncommon degree, so as to excite considerable alarm, even at a time when the pulse and other symptoms did not lead the practitioner to form an unfavourable opinion of the result.

In some cases convulsive motions of the face and of the different extremities have occurred, the former approaching to the disease called the risus sardonicus, and the latter accompanied with a kind of spasm of the fingers.

When these appearances have been observed, the fatal catastrophe has soon succeeded, much sooner indeed than might have been expected from merely observing the state of the pulse, and other indications of debility.

